# Assessment of user voluntary engagement during neurorehabilitation using functional near-infrared spectroscopy: a preliminary study

**DOI:** 10.1186/s12984-018-0365-z

**Published:** 2018-03-23

**Authors:** Chang-Hee Han, Han-Jeong Hwang, Jeong-Hwan Lim, Chang-Hwan Im

**Affiliations:** 1 Department of Biomedical Engineering, Hanyang University, Seoul, 04763 South Korea; 2 Kumoh National Institute of Technology, Department of Medical IT Convergence Engineering, Gumi, 38530 South Korea

**Keywords:** Functional near-infrared spectroscopy (fNIRS) - motor rehabilitation - neurorehabilitation - combined exercise - pattern classification - hemodynamic response

## Abstract

**Background:**

Functional near infrared spectroscopy (fNIRS) finds extended applications in a variety of neuroscience fields. We investigated the potential of fNIRS to monitor voluntary engagement of users during neurorehabilitation, especially during combinatory exercise (CE) that simultaneously uses both, passive and active exercises. Although the CE approach can enhance neurorehabilitation outcome, compared to the conventional passive or active exercise strategies, the active engagement of patients in active motor movements during CE is not known.

**Methods:**

We determined hemodynamic responses induced by passive exercise and CE to evaluate the active involvement of users during CEs using fNIRS. In this preliminary study, hemodynamic responses of eight healthy subjects during three different tasks (passive exercise alone, passive exercise with motor imagery, and passive exercise with active motor execution) were recorded. On obtaining statistically significant differences, we classified the hemodynamic responses induced by passive exercise and CEs to determine the identification accuracy of the voluntary engagement of users using fNIRS.

**Results:**

Stronger and broader activation around the sensorimotor cortex was observed during CEs, compared to that during passive exercise. Moreover, pattern classification results revealed more than 80% accuracy.

**Conclusions:**

Our preliminary study demonstrated that fNIRS can be potentially used to assess the engagement of users of the combinatory neurorehabilitation strategy.

## Background

Patients with motor impairments resulting from various central nervous system diseases such as stroke, cerebral palsy, and Parkinson’s disease encounter several difficulties with their activities of daily life. Those severely affected are unable to perform even basic body movements without the help of caregivers or assistive devices. The motor impairments consistently compromise the quality of their life [1].

The most common neurorehabilitation strategy for the recovery of impaired motor function is the repeated movement of the body parts associated with the damaged brain areas. The positive effect of this classical rehabilitation approach on motor re-learning has been demonstrated by a large number of previous studies. For example, some animal studies have shown that the continuous repetition of body movements can lead to both structural and functional enhancements of the motor cortex [2–5]. Human studies have also confirmed that the repetitive movement of impaired limbs can result in the improvement of affected motor functions [6–8]. In conventional motor rehabilitation interventions, patients do not voluntarily perform the repetitive movement of their impaired body parts, but the movement is enforced by a physiotherapist or an assistive rehabilitation device. This form of neurorehabilitation is called the passive exercise (PE) strategy. Although PE can lead to enhancement of motor performance, its effect is limited compared to the so-called active exercise (AE) strategy that involves voluntary movements of the patients [9, 10]. To enhance the clinical outcome of motor rehabilitation, several studies have recently proposed advanced rehabilitation strategies involving the combination of PE with AE, called a combinatory exercise (CE) strategy. In this rehabilitation approach, patients are required to be voluntarily engaged in rehabilitation training by additionally performing active motor execution (AME) while passive motor execution (PME) is involuntarily conducted by a physiotherapist or an assistive rehabilitation device. In the case that an affected part is rendered immovable, motor imagery (MI), a mental rehearsal of specific motor acts without overt movement, can be used instead of AME.

Gritsenko et al. first introduced a CE approach in which hand open was passively performed by functional electrical stimulation (FES) while the other movements for performing the given motor tasks were performed by the experimental patients without any assistance [11]. This study showed that the proposed CE approach could effectively improve hand functions in patients with hemiplegia. Since the study of Gritsenko et al., researchers have investigated the relationship of the motor functions enhanced by CE to brain activity changes in the motor cortex using neuroimaging modalities such as functional magnetic resonance imaging (fMRI) [12] and electroencephalography (EEG) [13]. The two studies [12, 13] showed that performing CE (PME + AME or PME + MI) was more effective in restoring impaired motor functions than PE alone, and further confirmed that CE is more effective in augmenting brain activity around motor areas compared to PE alone.

The main purpose of CE-based rehabilitation approaches is the active involvement of patients during PME tasks that are usually carried out by therapists or rehabilitation devices. In the current CE-based rehabilitation programs, however, it is nearly impossible to directly assess whether the patients are actively engaged in the given motor tasks especially when the movement of an impaired part of the body is made by an external neurorehabilitation device such as rehabilitation robots and FES. Thus, continuous verbal instructions may be the only option to actively and consistently involve the patients in the current CE-based rehabilitation exercises. If therapists could clearly determine the involvement of the patients during CE, it would help them provide timely feedbacks to patients. This could significantly improve the efficiency of the CE-based rehabilitation programs. To the best of our knowledge, however, the quantitative evaluation of active involvement of patients in given motor tasks during CE has not been accomplished.

The fundamental goal of the present study was to assess the degree of engagement of users of CE-based rehabilitation programs, based on the differences in neurophysiological findings of the activated motor-related brain areas during combinatory movement (PME + AME or PME + MI), compared to PME alone. In this study, fNIRS was used to measure brain activity because it has been well documented that fNIRS is less sensitive to motion artifacts [14] and the changes in cognitive states such as attention [14] and vigilance [15] can be successfully decoded from the fNIRS signals. Eight healthy participants were recruited in this study and were administered three different rehabilitation exercises, one based on PE (PME only) and two on CEs (PME + MI and PME + AME), during which hemodynamic responses were measured using a multi-channel fNIRS system. We performed statistical analyses to examine the differences of hemodynamic responses during the three experimental conditions, and attempted to classify the respective hemodynamic responses (PME vs. PME + MI and PME vs. PME + AME) to determine the accuracy of identification of voluntary engagement of the users using fNIRS signals.

## Methods

### Participants

Eight healthy participants (6 men and 2 women, average age 26.13 ± 2.23 years, all right handed) were enrolled in the present study. None of them had a previous history of neurological, psychiatric, or other severe diseases that could affect the experimental results. A comprehensive summary of the experimental procedure and protocol was provided to each subject before starting the experiment. They provided informed consent and were reimbursed for their participation on completion of the experiment. The study was reviewed and approved by the Institutional Review Board (IRB) committee of Hanyang University.

### Functional near-infrared spectroscopy (fNIRS) setup

**Fig. 1:**
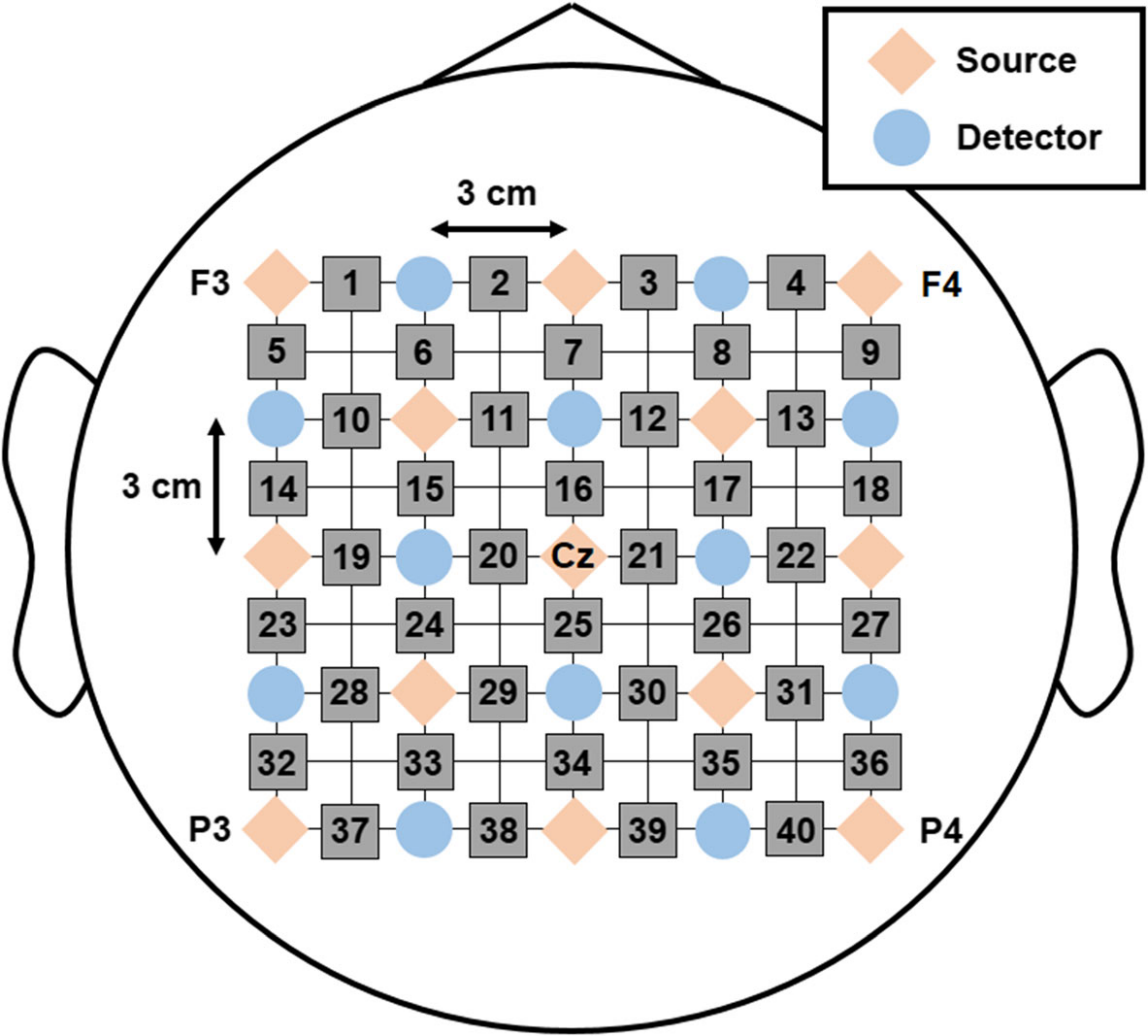
The configuration of optical probes. Red diamonds and blue circles illustrate position of source and detector of fNIRS system, and gray squares indicate position of channels. Distance between a source and detector was 3 cm

We used a commercial multi-channel fNIRS instrument (FOIRE-3000; Shimadzu Co. Ltd., Kyoto, Japan) for recording cortical hemodynamic activity. The system employs near-infrared lasers of three different wavelengths, 780, 805, and 830 nm. The distance between the source and detector was set at 3 cm, which was adequate to detect changes of brain hemodynamic responses induced by motor execution or motor imagery [16]. The absorption rates of the three near-infrared lights with different wavelengths were acquired at a sampling rate of 10 Hz and transformed into concentration changes of oxygenated hemoglobin (oxy-Hb), deoxygenated hemoglobin (deoxy-Hb), and total hemoglobin (total-Hb) using the modified Beer-Lambert law [17]. The sources and detectors were placed on the scalp surface using an elastic cap according to the international 10–20 system that is the standard method for electrode attachment for EEG recording x[18]. In the present study, we used twelve sources and thirteen detectors, resulting in 40 NIR channels, as shown in Fig. 1. Most optodes were distributed around the motor cortex, thereby covering the premotor area, supplementary motor area (SMA), primary motor area (M1), and posterior parietal cortices. The center source was located at Cz position (see Fig. 1). Prior to the experimental recordings, we confirmed that the 40 channels functioned appropriately in terms of light intensity.

### Experimental paradigm

**Fig. 2:**
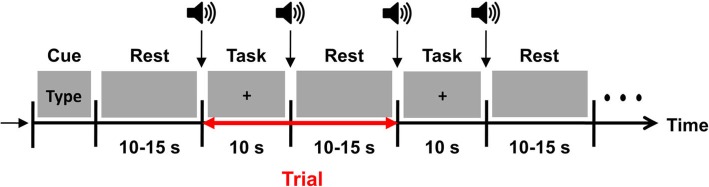
A schematic diagram of the experimental paradigm. At the beginning of the experiment, instructions for one of the three types of tasks, that is either, PME, PME + MI, or PME + AME appeared on the LCD monitor. One trial consisted of a randomized inter-task rest period ranging from 10 to 15 s and a task period of 10 s. A short beep sound followed before task and rest period

During the experiment, the participants were seated in a comfortable armchair, facing a 17-in. LCD monitor, which provided the instructions for the experimental tasks. The distance between the participant and the LCD monitor was set at 50 cm. At the beginning of the experiment, an instruction for one of the three task types (PME, PME + MI, or PME + AME) appeared on the LCD monitor for 5 s, after which the participants were given a variable rest period (10–15 s) to prepare for the given task. The participants performed the designated task for 10 s immediately after a short pure-tone beep sound, which was also used as a prompt to fix their gaze at a fixation cross at the center of the monitor to prevent any potential loss of concentration. This procedure was repeated twenty times in a session, as shown in Fig. 2. Each participant was administered six sessions in total, two sessions (40 trials = 20 trials × 2 sessions) for each of three tasks. The order of the tasks was randomly determined. The following paragraphs provide detailed descriptions of the three tasks.

**Fig. 3:**
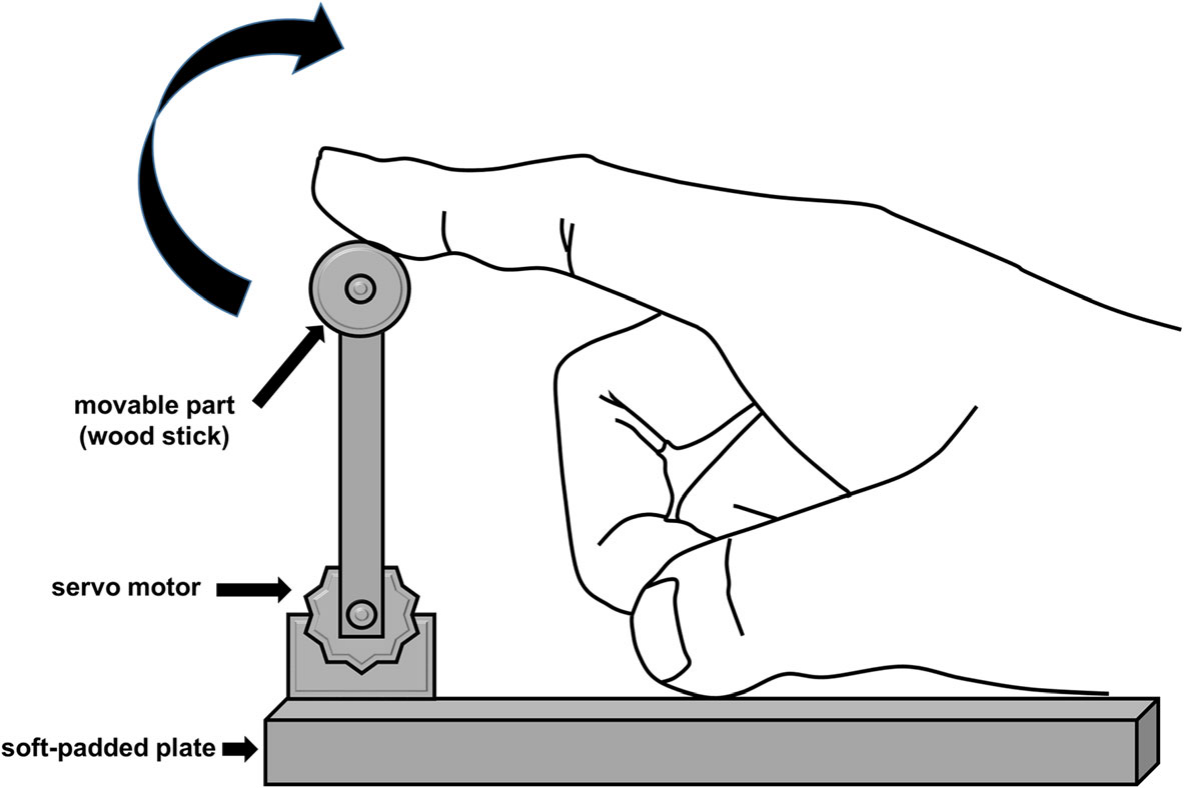
Illustration of the in-house hardware system developed by the authors for this study

- *PME* - The right index finger of the subject was automatically moved by an in-house hardware system that was designed to enforce the bending and stretching of a finger with a constant speed of 1 Hz (see fig. 3 for the schematics of the system). During the task, the participant was asked not to perform either MI or AME.
- *PME + MI* - The participants simultaneously performed both PME and MI. They imagined tapping the right index finger with the same speed as that enabled with the hardware system while the right index finger was automatically moved by the device. During the task period, they were asked not to perform voluntary motor execution of their right index finger (AME).
- *PME + AME* - The subjects performed both PME and AME using the right index-finger, with instructions similar to the PME + MI task, except for performing AME instead of MI. Note that the voluntary finger movement did not actually influence the operation of the device.

### Data preprocessing

The concentration changes of oxy-, deoxy-, and total-Hb were preprocessed using a series of signal processing algorithms to reduce physiological and environmental noise. First, we applied a common average reference (CAR) spatial filter to remove unwanted artifacts (e.g., artifacts due to heartbeat or respiration). Several NIRS studies have demonstrated that the CAR filter can effectively reduce the global influence of heartbeat or respiration [19–21]. After applying the CAR, the NIRS data were band-pass filtered using a fourth-order zero phase Butterworth filter with a pass-band of 0.01–0.1 Hz to reduce physiological noise and low-frequency drifts [22–27]. The filtered data were then segmented, including 10 s task period and the following 10 s rest period, considering that brain hemodynamic responses are inherently delayed several seconds compared to brain electrical activity. For baseline correction, we used a base reset method, which adjusts the first sample of each epoch to the zero point [28, 29]. Using the preprocessed data, we performed statistical analysis to reveal the characteristics of hemodynamic responses induced by the various experimental conditions (PME, PME + MI, and PME + AME), and pattern classification was applied to estimate the degree of active involvement of the participants during CE.

### Statistical analysis

Statistical analysis was carried out to investigate the differences between hemodynamic responses induced by PME alone and the two CEs (PME + MI and PME + AME). To construct the statistical dataset, each of 20 s epochs of oxy-, deoxy- and total-Hb was segmented using a moving window of 3 s with a 50% overlap for each channel. The mean of the hemodynamic responses in each segment was calculated, and then averaged over all epochs for each channel. This procedure was separately applied to each chromophore of NIRS (oxy-, deoxy, and total-Hb). Non-parametric Friedman test was performed because the test data set did not follow a normal distribution, and the Wilcoxon signed rank test with Bonferroni correction was conducted as a post-hoc analysis.

### Pattern classification

We conducted single-trial pattern classifications to investigate the feasibility of decoding the cognitive engagement of users during the CE. In particular, the NIRS data recorded during PME were distinguished from those recorded during PME + MI or PME + AME. Feature vectors for the classification were independently constructed for each of the three types of hemodynamic responses (oxy-, deoxy-, and total-Hb), and NIRS channels showing statistically significant difference between two conditions (PME vs. PME + MI or PME vs. PME + AME) in the above mentioned statistical tests were only considered (see Table 1 for the statistical analysis results).

**Table 1:** Detailed results of statistical analysis

Hb Type	Channel No.	Side	Time Period (sec)	Post-hoc Results
Oxy-Hb	3	ipsi	9 to 12	1 vs 2^*^ (+)
	13	ipsi	9 to 12	*1 vs 2^**^ (+)
	21	ipsi	0 to 30	1 vs 2^*^ (+)
	27	ipsi	9 to 12	1 vs 2^*^ (+)
	33	contra	6 to 9	*1 vs 2^**^ (+)
	34	central	6 to 9	1 vs 2^*^ (+), 1 vs 3^*^ (+)
	39	ipsi	9 to 12	*1 vs 2^*^ (+), 1 vs 3^**^ (+)
Deoxy-Hb	17	ipsi	9 to 12	*1 vs 2^**^ (−)
	17	ipsi	12 to 15	*1 vs 2^**^ (−)
	26	ipsi	0 to 3	1 vs 2^*^ (−)
	38	contra	3 to 6	1 vs 3^*^ (−)
Total-Hb	15	contra	6 to 9	1 vs 2^*^ (+), 1 vs 3^*^ (+)
	15	contra	9 to 12	*1 vs 2^*^ (+), 1 vs 3^**^ (+)
	16	central	6 to 12	1 vs 3^*^ (+)
	25	central	9 to 12	1 vs 3^*^ (+)
	27	ipsi	6 to 9	1 vs 2^**^ (+), 1 vs 3^**^ (+)
	27	ipsi	9 to 12	1 vs 2^**^ (+), 1 vs 3^**^ (+)
	31	ipsi	6 to 9	*1 vs 2^*^ (+), 1 vs 3^*^ (+)*
	31	ipsi	9 to 12	*1 vs 2^*^ (+), 1 vs 3^**^ (+)
	35	ipsi	6 to 9	*1 vs 2^*^ (+), 1 vs 3^**^ (+)
	35	ipsi	9 to 12	*1 vs 3^**^ (+)
	36	ipsi	9 to 12	1 vs 3^*^ (+)
	37	contra	0 to 3	*1 vs 2^**^ (+)
	38	contra	3 to 6	1 vs 2^*^ (+)
	39	ipsi	9 to 12	1 vs 3^*^ (+)
	40	ipsi	6 to 9	1 vs 2^**^ (+), 1 vs 3^*^ (+)*
	40	ipsi	9 to 12	1 vs 2^*^ (+), 1 vs 3^*^ (+)

*ipsi* ipsilateral hemisphere, *contra* contralateral hemisphere, *central* central area

1: PME, 2: PME + MI, 3: PME + AME;

*p-value < 0.05, **p-value < 0.01;

(+): A < B in A vs B, (−): A > B in A vs B;

Since the delay in task-related hemodynamic responses generally varies from 3 to 8 s [16, 22, 30, 31], different time windows with different sizes should be considered for effective determination of the delayed hemodynamic responses [32–34]. Thus, we used different moving window sizes of 1, 2, 4, 5, and 10 s with a 50% overlap, and then extracted five different features from each time window. The extracted five features were mean, variance, kurtosis, skewness, and slope of NIRS signals, which were noted as promising candidates for features in previous NIRS studies [23, 32, 35]. As large numbers of features may lead to over-fitting of a classifier, feature selection was performed using the Fisher’s score method that has been frequently used for NIRS-based pattern classification [23, 24, 27]. In this study, linear discriminant analysis, which has been successfully employed in several previous NIRS-based studies [24, 27, 32, 36], was used as a classifier. A 10 × 10 cross-validation was applied for the evaluation of classification accuracy.

In addition, we attempted to improve the determination of involvement of users during the CEs using a multiple-trial classification approach. For this, we applied a voting scheme to the same feature set extracted in the single-trial classification procedure. A voting scheme has been widely used for pattern classification, and shows enhanced performance in terms of classification accuracy [37–40]. In the voting method, multiple trials are conducted and a decision is made when a majority of test trials agree with a specific class. In this study, we applied a voting method with different numbers of test trials, which were three, five, seven, and eleven, for each class. For example, when the number of test trials was three, thirty-seven of forty trials were randomly selected for each class and used for building a classifier, and the remaining three trials were tested for each class. This procedure was iterated 1000 times, and classification accuracy was attained by averaging all the classification accuracies. The voting method was similarly applied for the other numbers of test trials (five, seven, and eleven).

### Performance evaluation

To validate the performance of the proposed approach, we calculated three different metrics: accuracy, sensitivity, and specificity. When PME + MI or PME + AME is assumed to be positive condition and PME is negative condition, the three metrics are defined as

$$ {\displaystyle \begin{array}{c}\mathrm{Accuracy}=\left(\mathrm{TP}+\mathrm{TN}\right)/\left(\mathrm{TP}+\mathrm{TN}+\mathrm{FP}+\mathrm{FN}\right),\\ {}\mathrm{Sensitivity}=\mathrm{TP}/\left(\mathrm{TP}+\mathrm{FN}\right),\mathrm{and}\\ {}\mathrm{Specificity}=\mathrm{TN}/\left(\mathrm{FP}+\mathrm{TN}\right),\end{array}} $$

where TP, TN, FP, and FN represent the numbers of true positives (correctly identified positive condition), true negatives (correctly identified negative condition), false positives (positive condition incorrectly identified as negative condition), and false negatives (negative condition incorrectly identified as positive condition), respectively.

## Results

### Comparison of hemodynamic responses induced by different conditions

**Fig. 4:**
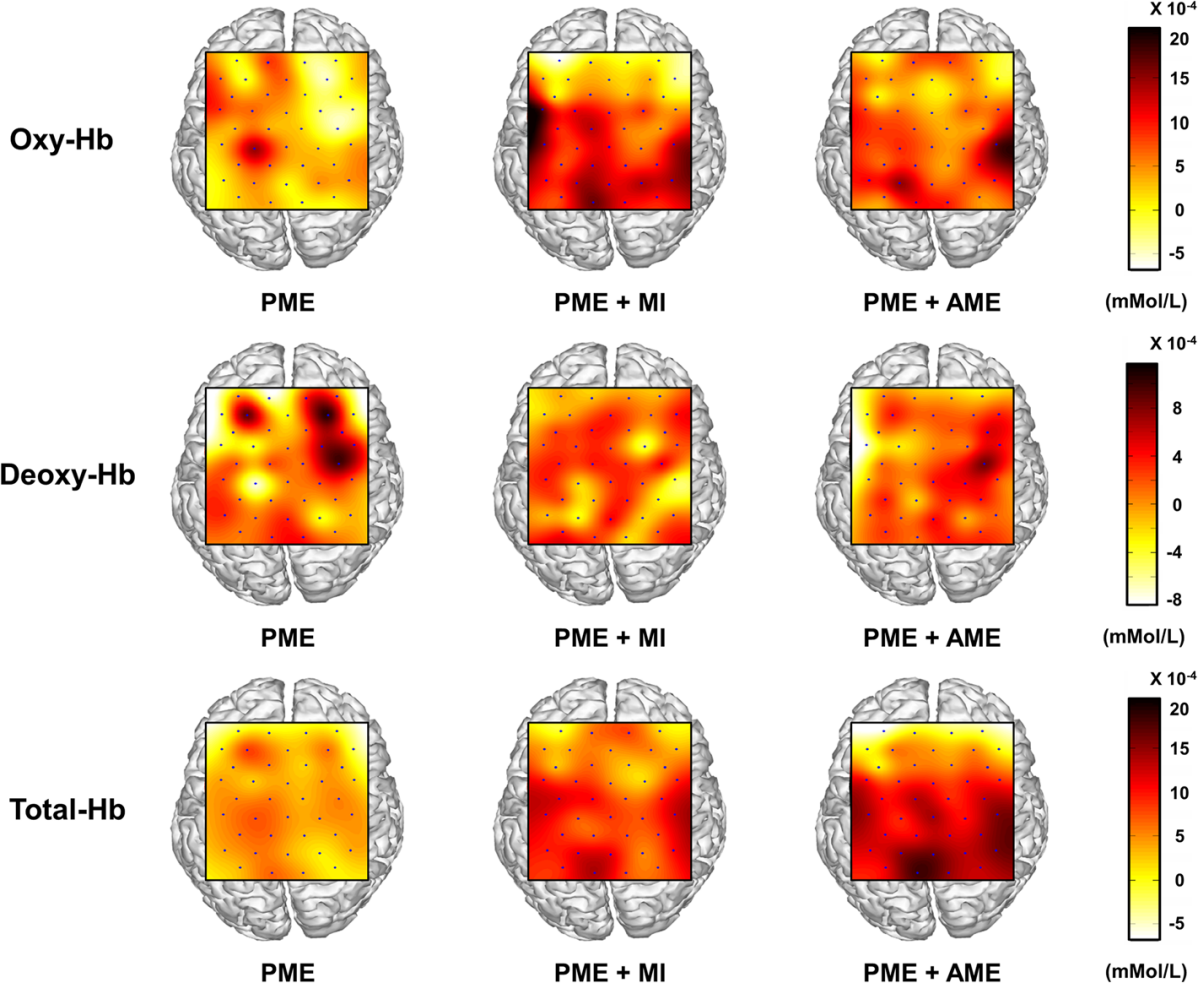
Topographic map of concentration change of oxy, deoxy-, and total-Hb for each task, such as passive exercise (PME), combinatory exercises (PME + MI or PME + AME). Blue points indicate the positions of channels

Figure 4 illustrates the grand-averaged topographic maps of oxy-, deoxy-, and total-Hb concentration changes for three different conditions. It is clearly observed from the figure that the modulation of hemodynamic responses with CE tasks (PME + MI and PME + AME) was broader and stronger than PME alone. In particular, increase in hemodynamic responses is observed around the bilateral motor areas during CE tasks, while hemodynamic responses induced by PME alone are localized in the contralateral motor cortex.

Table 1 shows the detailed results of the statistical analysis performed to investigate the difference between PME alone and CE tasks. Seven channels in oxy-Hb (ch. 3, 13, 21, 27, 33, 34, and 39), three channels in deoxy-Hb (ch. 17, 26, and 38), and eleven channels in total-Hb (ch. 15, 16, 25, 27, 31, 35, 36, 37, 38, 39, and 40) showed statistically significant differences among different conditions.

### Results of single- and multiple-trial pattern classification

**Fig. 5:**
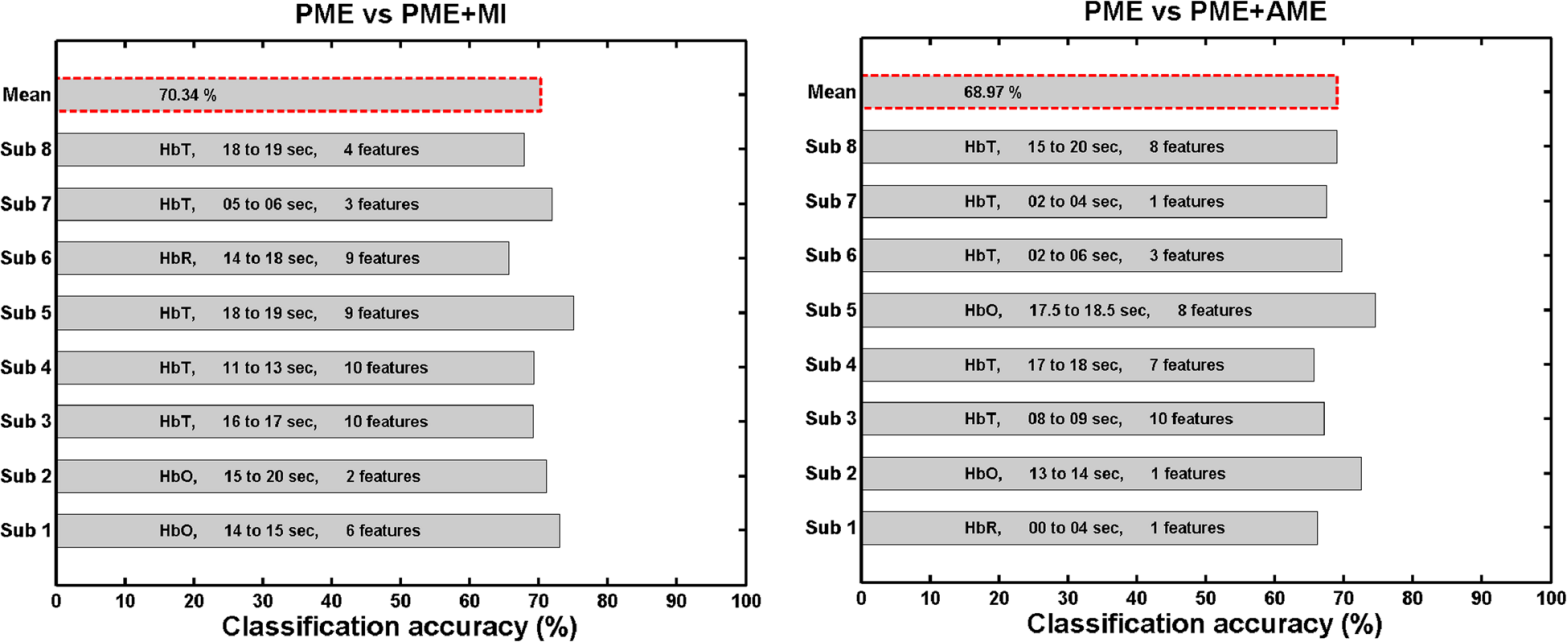
Individual classification accuracy of single-trial pattern classification. Bar with red dotted border shows average classification accuracy. PME vs PME + MI and PME vs PME + AME indicate classification PME versus PME + MI and PME versus PME + AME, respectively. The characters in each bar show best feature set used for each individual

Figure 5 shows the classification accuracies of each subject when “subject-specific” best feature set was used for the single-trial classification. PME and PME + MI conditions could be classified with an average classification accuracy of 70.34%. Of the eight subjects, four showed a classification accuracy of higher than 70%, which is marginal accuracy for determining the reliability of binary classification [41–43]. Similarly, PME and PME + AME could be classified with an average classification accuracy of 68.97%.

**Fig. 6:**
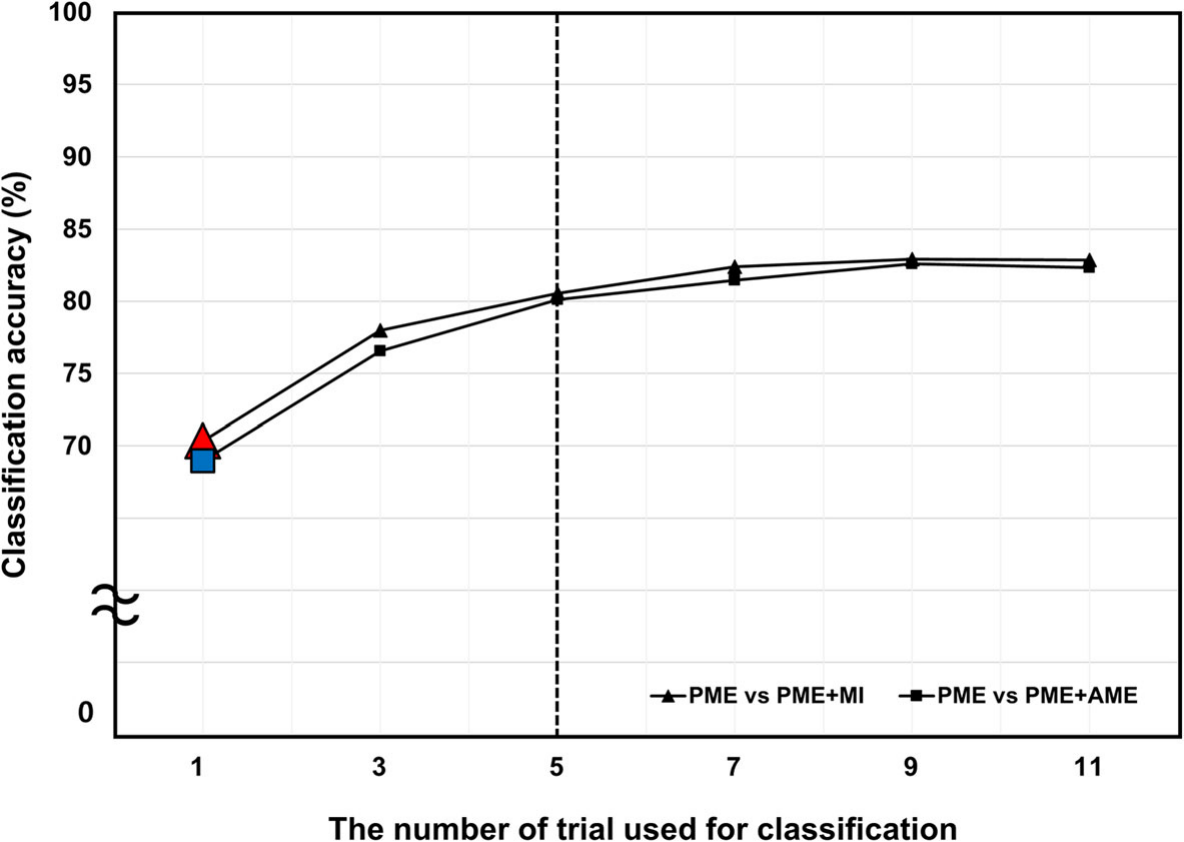
Classification accuracies with respect to the number of trials used for the classification. A black dotted vertical line indicates a saturation point. Red triangle and blue rectangle indicate average accuracies of single-trial pattern classification, respectively

Figure 6 shows the accuracy of multiple-trial classifications with respect to the number of test trials. As shown in the figure, higher classification accuracy was obtained with a higher number of trials. Nevertheless, the classification accuracy was almost saturated when more than five test trials were used. Table 2 shows the individual classification accuracies when five trials were tested. The average classification accuracies were 80.55% for PME vs. PME + MI and 80.11% for PME vs. PME + AME.

**Table 2:** Result of multiple-trial pattern classification

Subject No.	PME vs PME + MI			PME vs PME + AME		
	Accu (%)	Sens (%)	Spec (%)	Accu (%)	Sens (%)	Spec (%)
1	82.80	85.50	80.10	76.10	80.90	71.30
2	83.20	83.80	82.60	85.60	81.40	89.80
3	80.00	81.50	78.50	76.35	76.40	76.30
4	78.80	82.80	74.80	72.15	77.50	66.80
5	84.50	95.70	73.30	89.25	90.20	88.30
6	76.70	81.20	72.20	82.05	76.10	88.00
7	80.65	92.10	69.20	79.40	83.20	75.60
8	77.75	79.50	76.00	79.95	78.00	81.90
Mean	80.55 ± 2.77	85.26 ± 5.70	75.84 ± 4.40	80.11 ± 5.50	80.46 ± 4.69	79.75 ± 8.58

*Accu* Accuracy, *Sens* Sensitivity, *Spec* Specificity

## Discussion

### Feasibility of monitoring active engagement during CE

During the neurorehabilitation procedure, active participation of patients is a crucial factor to foster better recovery of the damaged brain region [44]. It is important to assess whether patients are actively engaged in the given neurorehabilitation programs. If therapists can accurately monitor the change of cognitive engagement during the rehabilitation program, the effect of rehabilitation can be improved as timely feedback can be provided to the patient. Unfortunately, however, methods to quantitatively assess active participation of the user during CE have rarely been developed. To the best of our knowledge, only one study with EEG has demonstrated that active movement can induce larger event-related desynchronization (ERD) than passive movement [45]. The study classified EEG data recorded during active and passive motor tasks using features derived from ERD with an average classification accuracy of about 80%. The authors insisted that EEG might be used to assess cognitive engagement during motor rehabilitation in stroke patients. Although the study reported the feasibility of evaluating the cognitive engagement of the user during the rehabilitation program by classifying PME and AME, it did not estimate the active involvement of the user during the CE rehabilitations. In this study, we have investigated whether the voluntary involvement of the user during CEs can be identified using NIRS. Our experimental results showed that the PME alone and CE tasks could be classified with a fairly high classification accuracy, slightly higher than 80%, using a multiple-trial classification (see Table 2). A previous EEG study [45] reported sensitivity, specificity and accuracy values of 83.55%, 80.16%, and 81.82%, respectively, in classifying PME and PME + MI. Although the motor task used in the previous EEG study was different from that used in our study, the performance metrics of our approach (sensitivity: 85.26%, specificity: 75.84%, and accuracy: 80.55%) were comparable to those of the EEG study, suggesting that NIRS can also be used to assess the user’s cognitive engagement during the motor rehabilitation program with CE tasks.

### Hemodynamic responses during CE tasks

Some of the previous studies have reported brain activation induced by CE tasks using different neuroimaging modalities [12, 13] such as fMRI and EEG. In an fMRI study, Joa et al. reported an increment of brain activation in the SMA, M1, primary somatosensory area (S1), secondary somatosensory area (S2), and cerebellum during CE while simultaneously performing PME and AME. An EEG study [13] also reported stronger brain activations in bilateral sensorimotor areas and the SMA during active than passive tasks. In the present study, we observed the hemodynamic responses during the CE tasks using NIRS. The hemodynamic responses of the CE tasks were significantly broader and stronger than those of the PME alone (see Fig. 4 and Table 1), which is in line with the results of the previous fMRI and EEG studies [12, 13]. Specifically, hemodynamic responses were increased in the ipsilateral as well as contralateral areas during CE tasks, which is also in agreement with the results of some of the previous studies that showed bilateral brain activations during MI or AME [9, 46–48].

### Limitations and future prospects

One of the main goals of this study was to confirm whether there is significant difference in hemodynamic responses induced by PE and CE using NIRS. Thus, we recruited eight healthy participants and analyzed hemodynamic responses acquired during the course of the rehabilitation program based on CE. In this study, patients with brain lesions were not involved. As different brain activation patterns can be observed in patients with stroke or other central nervous system diseases [49, 50], experiments with patients need to be conducted in future.

This study was also carried out to confirm the feasibility of assessing the cognitive engagement of users using NIRS during CE-based rehabilitation. Although we could obtain feasible classification accuracies, high enough to evaluate whether the user actively conducted MI or AME during CEs, online experiments need to be conducted in future to further prove the practical applicability of NIRS-based assessment of cognitive engagement during CE. Despite these limitations, our results are meaningful because we have demonstrated the feasibility of using fNIRS for evaluating the cognitive engagement of users during CEs for the first time.

## Conclusions

The main objective of this study was to confirm whether fNIRS can serve as a useful tool to assess cognitive engagement during motor rehabilitation programs based on CE. We observed significant differences between hemodynamic responses induced by the PME alone and CE tasks, and obtained meaningful classification performance, to identify whether users are actively involved in given motor tasks, using the induced hemodynamic responses. Our experimental results demonstrated that hemodynamic responses induced during CE tasks can be potentially used to identify the voluntary engagement of users during CE-based motor rehabilitation interventions, thereby providing useful feedback that can promote more active involvement of users.

## Abbreviations

AE: Active exercise
AME: Active motor execution
CAR: Common average reference
CE: Combinatory exercise
deoxy-Hb: Deoxygenated hemoglobin
EEG: Electroencephalography
ERD: Event-related desynchronization
FES: Functional electrical stimulation
fMRI: Functional magnetic resonance imaging
fNIRS: Functional near-infrared spectroscopy
IRB: Institutional Review Board
M1: Primary motor area
MI: Motor imagery
oxy-Hb: Oxygenated hemoglobin
PE: Passive exercise
PME: Passive motor execution
S1: Primary somatosensory area
S2: Secondary somatosensory area
SMA: Supplementary motor area
total-Hb: Total hemoglobin
